# Effects of Levodopa on Impairments to High-Level Vision in Parkinson's Disease

**DOI:** 10.3389/fneur.2020.00708

**Published:** 2020-07-17

**Authors:** Stephen Anderson, Elizabeth L. Stegemöller

**Affiliations:** ^1^Integrated Neuroscience Program, Iowa State University, Ames, IA, United States; ^2^Department of Kinesiology, Iowa State University, Ames, IA, United States

**Keywords:** line discrimination, object discrimination, facial discrimination, visual working memory, object rotation

## Abstract

Studies have reported that Parkinson's disease (PD) is associated with impairments on cognitive visual tasks. However, the effects of dopamine on cognitive vision remain equivocal. The purpose of this study was to examine performance on cognitive vision tasks in persons with PD and the effects of levodopa on these tasks. Fourteen individuals with PD and 14 age- and sex-matched healthy older adults completed the study. Participants with PD completed the visual tasks following a 12-h withdrawal of dopaminergic medication and again 1 h after taking 1.5 times their normal dose of levodopa. Healthy older adults completed the visual tasks twice using the same session format. Five complex visual tasks were completed, including line discrimination, object discrimination, facial discrimination, visual working memory, and object rotation. The Unified Parkinson's Disease Rating Scale was also collected off and on medication. Participants with PD performed significantly worse than the healthy older adults across all five visual tasks. There were no significant differences in performance between the off and on medication state in persons with PD. This finding indicates either that dopamine deficiency may not be responsible for cognitive visual impairments in PD or that cognitive visual impairments in PD might simply be the result of deficits in more basic visual processing.

## Introduction

A broad spectrum of visual symptoms has been observed in persons with Parkinson's Disease (PD). Indeed, 77% of individuals with PD report at least one recurring visual symptom, while 43% report two or more (1), but the research findings on visual processing impairments in PD, along with the effect of medication, remain equivocal. Thus, there is a need to better understand visual impairment in persons with PD.

Deficits in low level visual processing are evident in persons with PD, including impairments in visual acuity (2, 3), contrast sensitivity (4), and line discrimination (4–6). Impairments at these lower levels of visual processing may be affecting downstream visual processes such as object and face recognition, but evidence regarding the existence of high level visual processing deficits in PD is conflicting. While some studies have found that persons with PD have difficulty identifying objects (4), others have found minimal, if any, deficits in recognizing objects (7). Similarly, studies examining face recognition in persons with PD have found impairments (8, 9), while others have not (10). In some cases these deficits in object and facial recognition may be explained by impairments in visual working memory. Indeed, research has shown that persons with PD score significantly lower than HOAs on remembering novel faces for short durations (9). Finally, studies have indicated that PD is associated with impairments of mental rotation of objects (11), but others have failed to observe deficits in mental rotation (12).

Given the equivocal research on visual deficits in persons with PD, it is not surprising that the effect of dopamine on vision in PD also remains conflicting. Impairments in low level visual processing show limited response to treatment with levodopa (2, 3), and other symptoms such as dry eyes and inflammation of the inner eyelid may be more likely causes of these deficits (3). In high level visual processing, research is limited regarding the effects of dopaminergic medication. Dopaminergic medication has been shown to play a role in working memory more generally (13), but the effects on visual working memory remain unknown. There are limited reports on the effect of dopaminergic medication on either object or facial recognition and mental rotation.

Thus, the purpose of this study was to examine performance on cognitive vision tasks in persons with PD and the effects of levodopa on these tasks. Two aims were addressed. The first was to use any observed performance differences between individuals with PD and their healthy counterparts across tasks to determine where visual processing might be breaking down. The second was to determine the extent to which performance on visual tasks varies between ON-meds and OFF-medications states. Accordingly, five experiments were designed to assess cognitive vision that recruits processing resources at various levels. It is hypothesized that persons with PD will show differences in performance across all five visual tasks compared to HOAs, and that levodopa will improve performance.

## Materials and Methods

### Participants

Fourteen participants with a diagnosis of PD (Age: *M* = 71.1, *S.D*. = 5.8; five Female, nine Male; Education = 15.0 years) and 14 age- and sex-matched HOAs (Age: *M* = 70.1, *S.D*. = 4.9; five Female, nine Male; Education = 15.8 years) completed the study. The PD diagnosis was confirmed by the participant's treating neurologist or movement disorders specialist. The two groups did not differ significantly in age (*p* = 0.81) or years of education (*p* = 0.51). Inclusion criteria for all participants included normal or corrected-to-normal vision as determined by an eye appointment within the last 12 months. Additional inclusion criteria for participants with PD was that they were currently taking a stable dose of levodopa for at least the last 30 days. Exclusion criteria included evidence of cognitive impairment (Mini Mental Status Exam <24) and/or depression (Geriatric Depression Scale > 5). See Table 1 for participant demographics. All participants provided informed consent, and the study was approved by the Institutional Review Board at Iowa State University.

**Table 1:** Participant demographics.

**Healthy older adults**					**Individuals with Parkinson's disease**							
**ID**	**Gender**	**Age**	**Education (yrs)**	**Handedness**	**ID**	**Gender**	**Age**	**Education (yrs)**	**Handedness**	**Total UPDRS**	**MAS**	**Disease duration (yrs)**
103	F	65	18	R	1	F	64	14	R	15	Right	6
109	M	75	18	R	2	M	77	12	R	67	Right	10
114	M	74	12	R	3	M	77	16	R	27	None	4
104	M	72	18	L	4	M	70	14	R	24	Right	11
101	F	69	12	R	5	F	70	12.5	R	51	None	7
119	M	63	12	R	6	M	61	24	R	63	Right	13
110	M	64	14	R	7	M	62	12	R	33	Left	6
118	F	67	12	R	8	F	70	12	R	46	None	12
102	M	67	20	R	9	M	67	16	R	39	Left	7
120	F	76	20	R	10	F	77	12	R	58	Right	8
112	M	66	16	R	12	M	67	16	R	65	Right	8
117	M	75	16	R	13	M	77	20	R	56	None	4
116	M	70	18	R	14	M	73	14	R	31	Left	16
106	F	78	16	R	15	F	75	16	R	41	Left	10
Average:		70.07	15.86		Average:		70.5	15.04				

*ID, Identification; F, female; M, male; R, right; L, left; yrs, years; UPDRS, Unified Parkinson's Disease Rating Scale; MAS, more affected side*.

### Protocol

Both the HOAs and participants with PD completed two sessions of visual processing testing during the same day: a morning session (T1) and an afternoon session (T2). Both groups completed all five visual tasks once during each session, and a 1-h break was provided between the two sessions. Prior to T1, participants with PD were asked to abstain from their morning dose of all parkinsonian medications, resulting in a 12-h withdrawal from medication. HOAs did not abstain from any medications, and participants with PD did not abstain from any non-Parkinsonian medications. After T1 ended, participants with PD were asked to take 1.5 times their standard dose of levodopa while continuing to abstain from any other antiparkinsonian medications. T2 started 1 h after taking the medication. HOAs did not take any medication after T1.

For both T1 and T2 sessions, all participants completed five visual processing tasks. Prior to completing the five tasks, all participants completed a computerized task designed to help them acclimate to their surroundings and familiarize them with the format of the experimental tasks. Each of the 20 trials in the acclimation task consisted simply of the simultaneous presentation of two photographic images on a computer screen, which were either family photographs or landscapes with no people present. Participants were asked to indicate whether the two images were the same or different by pressing one of two keys on a standard keyboard. Images in the “Same” condition were always identical, and images in the “Different” condition always consisted of one family photo and one landscape to maximize ease of judgement. Participants were given as long as they needed to make a decision on each trial, and whichever keys on the keyboard corresponded to “same” and “different” (either *z* or *m*) remained constant for all tasks for the remainder of the study.

Once they had completed the acclimation task, participants proceeded to complete all five of the computerized visual tasks. Due to the number of trials in Experiment 1, this task was divided into three parts with each administered separately. The three parts of the Experiment 1 task and the other four experiment tasks were all pooled and administered in random order. For the T2, all participants completed the same visual tasks from T1, except the acclimation task. All tasks were again administered in random order.

Prior to completing the visual tasks during T1, all participants with PD completed the motor portion of the Unified Parkinson's Disease Rating Scale (UPDRS). After the visual tasks were completed in T2, the full version of the UPDRS was completed by the PD group. The total score was used as an index of their overall disease progression and is shown in Table 1. The motor scores were used as an index of the effect of their levodopa medication. The UPDRS was scored by a trained rater.

### Visual Tasks Description

**Figure 1:**
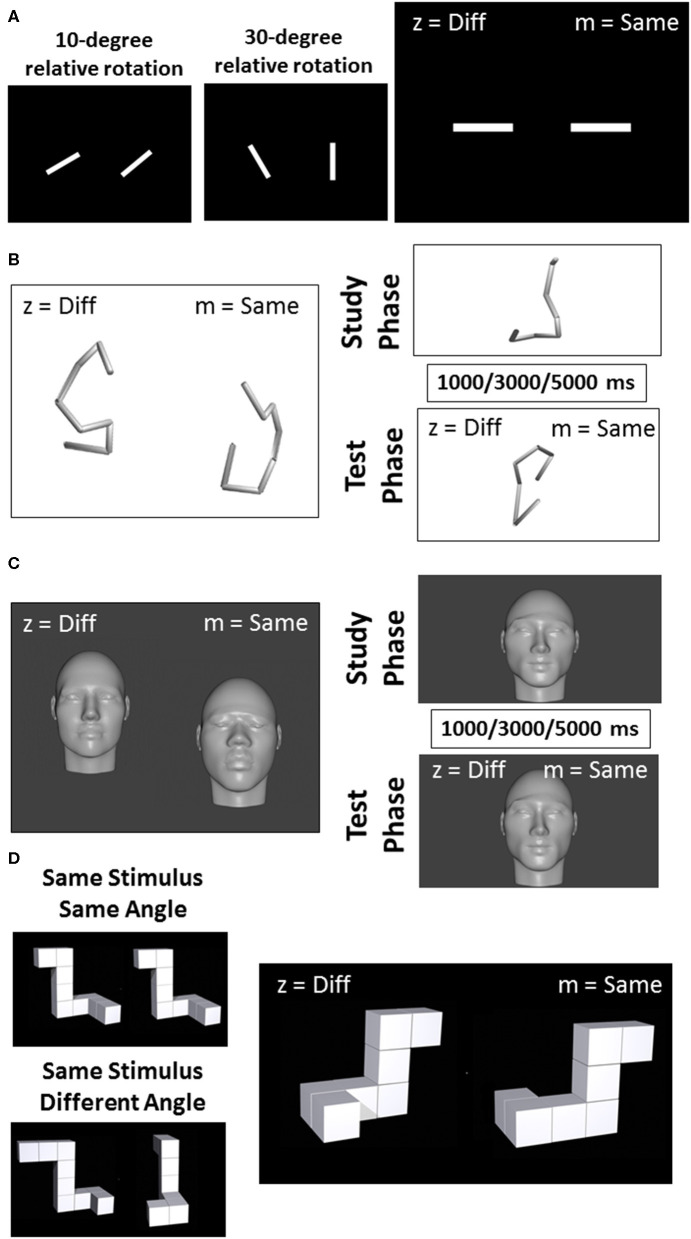
Example of visual stimuli used for the line discrimination task **(A)**, the object discrimination task **(B)**, the facial discrimination task **(C)**, and the object rotation task **(D)**.

All visual tasks have been used in previous research (14–16). Task 1, Line Discrimination, consisted of pairs of white line segments set against a black background (Figure 1A). Each trial began with a blank screen that persisted for 1 s, followed by the simultaneous presentation of two white horizontally aligned line segments. On each trial, one line segment was rotated in the picture plane between 0 and 90° from horizontal by some multiple of 10 (e.g., 10, 20, 30°, etc.). On half of the trials, the second line segment was at the same angle as the first (i.e., rotated at 0° with respect to the first segment) while on the other half of trials the second line segment was rotated between 10 and 90° with respect to the first, again by some multiple of 10°. The task consisted of 180 trials, but was administered in three installments of 60 trials each to minimize any effects of fatigue or proactive interference. Each of the three installments of this task was divided into blocks of 15 trials, between which the experiment paused and encouraged participants to take a break for as long as they liked prior to resuming.

The second task, Object Discrimination, began with a blank screen that persisted for 1 s, followed by the simultaneous presentation of two tube sculptures slightly offset about the horizontal axis (Figure 1B). The stimuli for task two were modeled on a set utilized by Logothetis et al. (14) and were created as a series of eight points that would fall within the surface of a cylinder measuring 20 units high and 10 units in diameter (i.e., all were equidistant from a central, vertical axis). These eight points were connected in serial order to form a figure composed of seven line segments. The set of points was constrained so that line segments formed by connecting them would be limited to between 4 and 10 units, and angles formed by adjacent segments were limited to between 90 and 120°. Once each set of points was calculated and drawn using a Python script, they were rejected if the resulting figures contained line segments that intersected, or if the figures were not sufficiently tall (i.e., at least 16 units on the vertical axis). Once each set of points had been finalized, they were rendered in MatLab2016b (https://www.mathworks.com/products/matlab.html). Conversion of these 3D-rendered images into.png files suitable for ePrime 2.0 was accomplished using a free open source graphics program called Blender (https://www.blender.org/). Seventy-two trials were completed in four equal-sized blocks with rest in between each block.

The procedure for task three was identical to task two except for the stimuli used. For this Facial Discrimination task, stimuli were generated from a series of photographs of human faces collected from Google images (Figure 1C). All photographs were required to have closed mouths and neutral expressions. Half of these photos were of men and half were of women. While the rendering process likely obscured any features that would have facilitated visual categorization into demographic groups, effort was made to ensure that the initial images represented a broad (though incomplete) cross section of individuals. Thus, for each gender category, photographs were further selected on the basis of race and region of origin so that the full stimulus set contained equal numbers of Asian, Australian Aboriginal, Black, Caucasian, Latina/o, and Middle Eastern individuals. Once these photos were collected, a computer program called FaceGen (https://facegen.com/) was used to render each face onto a 3-dimensional head. This process removed all of the color and hair from the faces so that each could be distinguished only through inspection of its structural features. The process also allowed all heads to be rendered at a constant height, so that they could not be distinguished on the basis of how closely their edges came to the border of the screen. Once these 3-dimensional heads were rendered, they were exported as.obj files and opened in Blender (http://www.blender.org), a freeware 3D art program that allowed for precise manipulation of the heads in 3-dimensional space. Seventy-two trials were completed in four equal-sized blocks with rest in between each block.

For the fourth task, Visual Working Memory, face and object stimuli were generated by the same methods used in tasks two and three, but new sets of these stimuli were created for this experiment, such that no face or tube sculpture was reused across tasks or sessions (Figures 1B,C). This task was composed of 72 trials. Each trial in this task comprised a study period and a test period. During the study period (2,500 ms), participants were asked to study and remember an object or face presented on the screen. The study period was followed by one of the three designated delay intervals. One-third of the trials had a 3,000 ms delay, while another one-third had a 1,000 ms delay, and another third had a 5,000 ms delay. The delay was followed by a test period in which participants were asked to indicate whether the object or face that subsequently appeared on the screen was the same as or different than the stimulus presented in the study period. Seventy-two trials were completed in four equal-sized blocks with rest in between each block.

The stimuli used in task five, Object Rotation, were a set of perspective line drawings of novel 3-dimensional objects (Figure 1D). This stimulus set was developed by Ganis and Kievit (15) as an update to a classic stimulus set developed by Shepard and Metzler (16). The objects consisted of 7–11 cubes serially connected face-to-face in such a way that four 90° angles were formed along the length of the object. On each trial, two of the line objects were simultaneously presented on the screen, aligned horizontally. On half of all trials, both stimuli had the same structural description, while on the other half one of the four angles were changed in orientation to slightly alter the figure. Further, stimuli were rotated with respect to one another by either 0, 50, 100, or 150° about the Y-axis. This task was composed of 36 trials delivered in four equal-sized blocks with rest in between each block.

For all tasks, half of all participants pressed the “z” key if they believed that the two visual stimuli were the same and pressed the “m” key if they believed the two visual stimuli were different, while the other half of participants received the reverse key mapping. There was no pre-set time limit on each trial, so the stimuli remained on the screen until the participants responded.

### Data Analysis

For each task, the percent correct was determined. For Task 1 (Line Discrimination), a 2 (Group: PD vs. HOA) × 2 (Session: T1 vs. T2) × 3 [Line Angle: Small (10, 20, and 30°), Medium (40, 50, and 60°), and Large (70, 80, and 90°)] mixed factorial ANOVA was completed. For tasks 2 and 3 (Object and Facial Discrimination, respectively) the effects of PD pathology and levodopa treatment were examined using a 2 (Group: PD vs. HOA) × 2 (Session: T1 vs. T2) ANOVA. For task 4 (Visual Working Memory), the effects of PD pathology and levodopa treatment on visual working memory were examined using a 2 (Group: PD vs. HOA) × 2 (Session: T1 vs. T2) × 2 (Stimulus Type: Objects vs. Faces) × 3 (Delay: 1,000, 3,000, and 5,000 ms) mixed factorial ANOVA. For task 5 (Object Rotation), the effect of PD pathology and levodopa treatment on mental rotation were examined using a 2 (Group: PD vs. HOA) × 2 (Session: T1 vs. T2) × 2 (Identity: Same vs. Different) × 4 (Angle: 0, 50, 100, and 150) mixed factorial ANOVA. *Post-hoc* analyses were completed with Tukey's Honestly Significant Difference tests, and significance was set at α = 0.05.

For the motor UPDRS, a within subjects *t*-test was completed to determine if there was an effect of medication on clinical motor symptoms in the participants with PD. In addition, difference scores for all tasks (afternoon session scores minus morning session scores) and for the motor portion of the UPDRS were calculated. Pearson Correlations were completed to examine the relationship between levodopa administration and task performance in persons with PD. Significance was set at α = 0.05.

## Results

**Figure 2:**
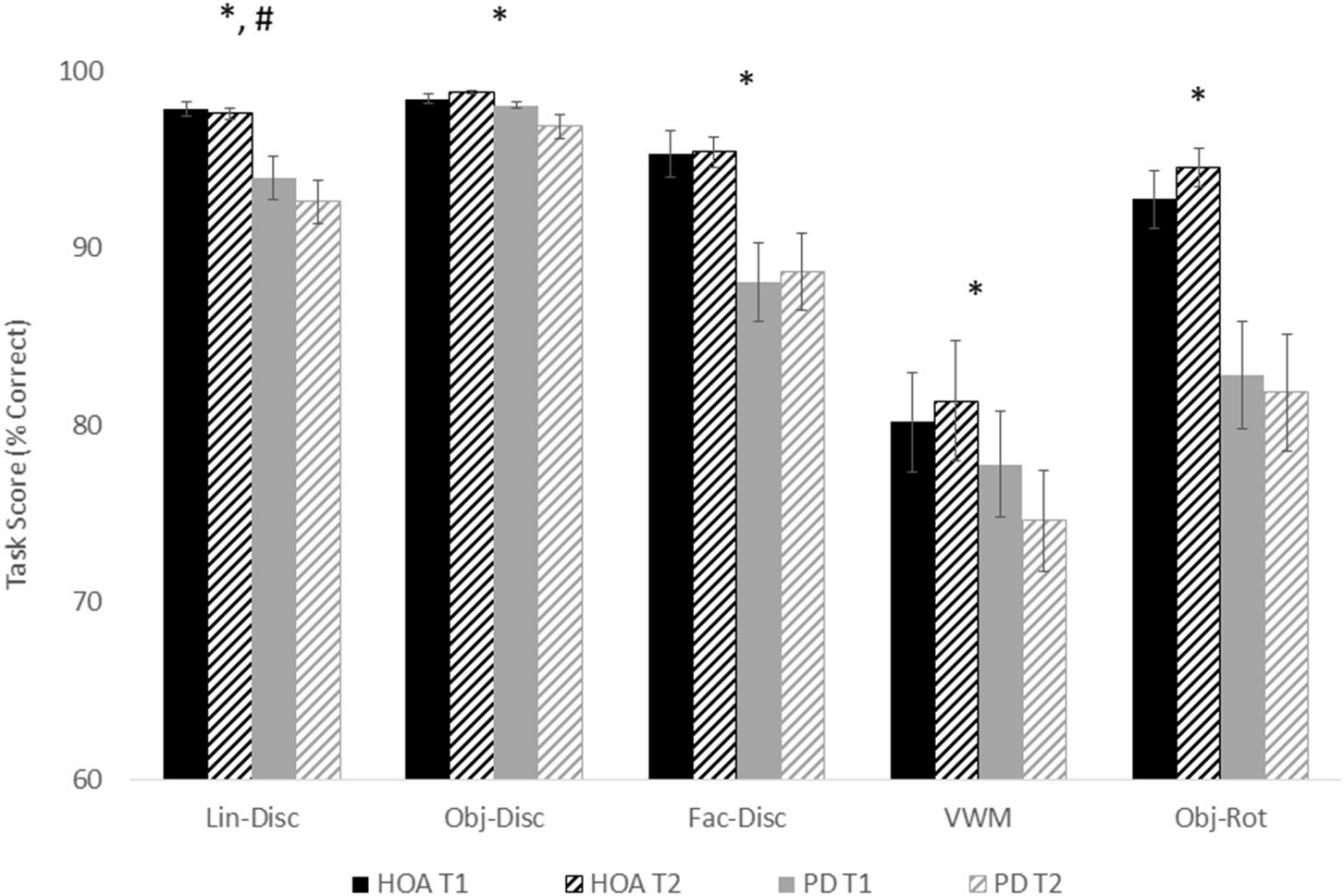
Mean and standard error for all tasks for both groups and for both sessions. Asterisks designate a significant group effect at *p* < 0.05, and the pound sign indicates a significant session effect at *p* < 0.05.

Figure 2 shows the mean and standard error for both groups for both sessions across each task. Across tasks, participants with PD performed worse than the HOAs. However, results did not differ between sessions, except for line discrimination. This would indicate that, in general, for participants with PD, medication did not affect performance on these tasks. Statistical results are reported below for each task (Table 2).

**Table 2:** Statistical results.

**Task 1:**	***F***	**df**	***p***	**Partial eta squared**	**Observed power**
Session	5.241	1	0.030	0.168	0.597
Group	13.451	1	0.001	0.341	0.942
Angle	23.491	2	0.000	0.475	1.000
Session × Group	4.013	1	0.056	0.134	0.488
Session × Angle	3.151	2	0.051	0.108	0.580
Group × Angle	4.338	2	0.018	0.143	0.728
Session × Group × Angle	1.150	2	0.324	0.042	0.242
**Task 2:**	***F***	**df**	***p***	**Partial eta squared**	**Observed power**
Session	1.680	1	0.206	0.061	0.239
Group	6.369	1	0.018	0.197	0.681
Session × Group	5.648	1	0.025	0.178	0.629
**Task 3:**	***F***	**df**	***p***	**Partial eta squared**	**Observed power**
Session	0.079	1	0.781	0.003	0.058
Group	10.708	1	0.003	0.292	0.883
Session × Group	0.040	1	0.843	0.002	0.054
**Task 4:**	***F***	**df**	***p***	**Partial eta squared**	**Observed power**
Session	0.072	1	0.791	0.003	0.058
Group	4.619	1	0.041	0.151	0.544
Stimulus type	224.202	1	0.000	0.896	1.000
Delay interval	6.976	2	0.002	0.212	0.912
Session × Group	0.347	1	0.561	0.013	0.088
Session × Stimulus type	0.118	1	0.734	0.005	0.063
Session × Delay interval	0.565	2	0.572	0.021	0.139
Group × Stimulus type	2.682	1	0.114	0.094	0.351
Group × Delay interval	0.259	2	0.772	0.010	0.089
Stimulus type × Delay interval	2.247	2	0.116	0.080	0.437
Session × Group × Stimulus type	0.209	1	0.651	0.008	0.073
Group × Stimulus type × Delay interval	0.159	2	0.853	0.006	0.073
Session × Group × Delay interval	1.306	2	0.280	0.048	0.270
Session × Stimulus type × Delay interval	0.274	2	0.761	0.010	0.091
Session × Group × Stimulus type × Delay interval	1.519	2	0.228	0.055	0.309
**Task 5:**	***F***	**df**	***p***	**Partial eta squared**	**Observed power**
Session	0.133	1	0.719	0.005	0.064
Group	12.043	1	0.002	0.334	0.914
Identity	0.125	1	0.727	0.005	0.063
Angle	3.245	3	0.027	0.119	0.723
Session × Group	1.401	1	0.248	0.055	0.206
Session × Identity	0.487	1	0.492	0.02	0.103
Session × Angle	1.871	3	0.142	0.072	0.466
Group × Identity	0	1	1.000	0	0.05
Group × Angle	0.428	3	0.734	0.018	0.131
Identity × Angle	1.114	3	0.349	0.044	0.289
Session × Group × Identity	0.92	1	0.347	0.037	0.151
Session × Identity × Angle	1.42	3	0.244	0.056	0.362
Identity × Angle × Group	0.382	3	0.766	0.016	0.122
Angle × Session × Group	0.652	3	0.584	0.026	0.181
Session × Group × Identity × Angle	0.339	3	0.797	0.041	0.113

**Figure 3:**
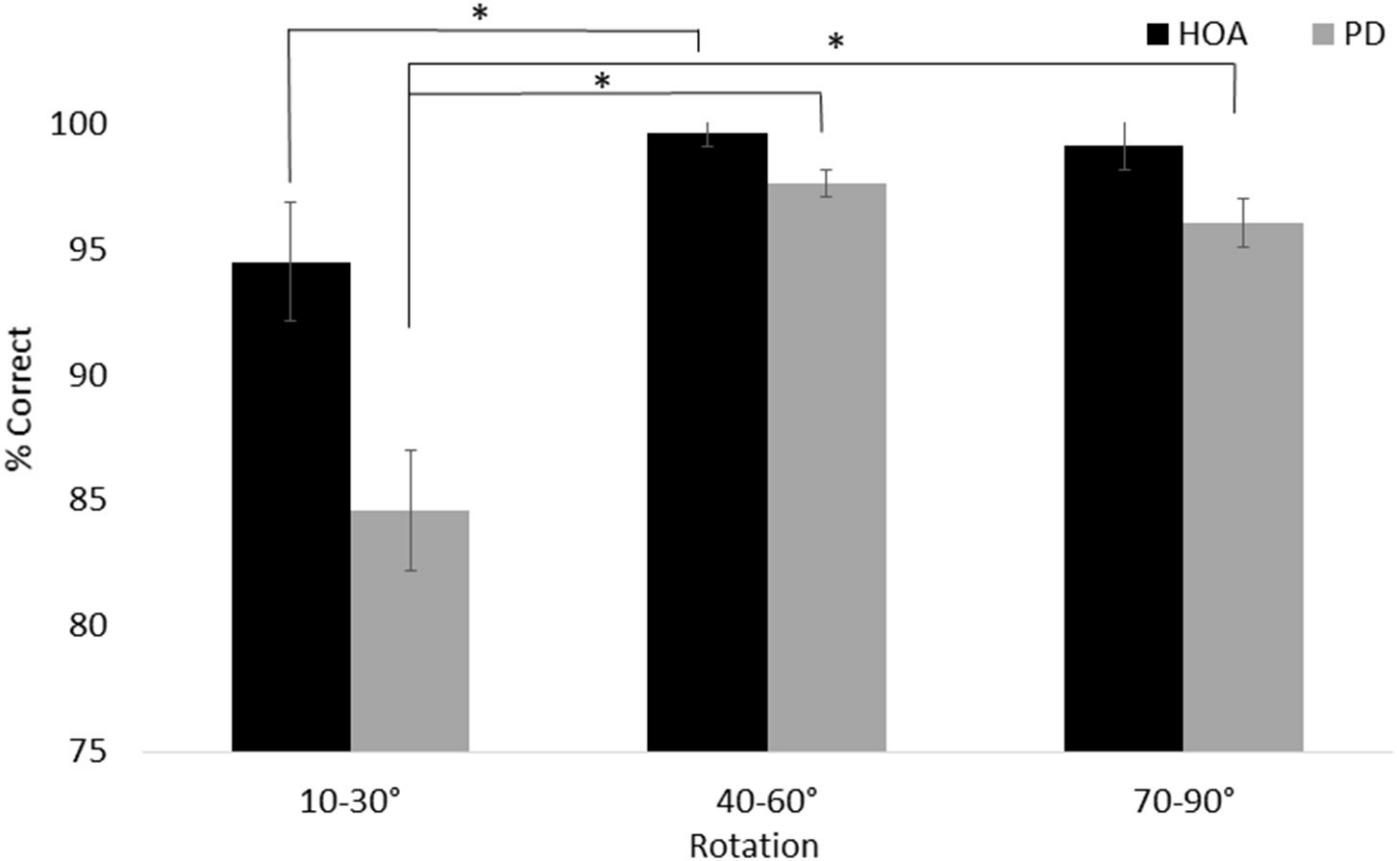
Mean and standard error for the line discrimination task. The interaction between group and angle is shown. Asterisks designate significance at *p* < 0.05.

For line discrimination (task 1), a main effect of group, *F*_(1, 26)_ = 13.45, *p* = 0.001, $\eta_{\text{p}}^{2}$ = 0.341, with the HOA group (*M* = 97.78%, *SE* = 0.96%) making more correct judgments than the PD group (*M* = 92.78%, *SE* = 0.96%) was revealed. There was also a main effect of session, *F*_(1, 26)_ = 5.24, *p* = 0.03, $\eta_{\text{p}}^{2}$ = 0.168, with participants across groups performing better in T1 (*M* = 95.91%, *SE* = 0.65%) than T2 (*M* = 94.64%, *SE* = 0.81%). Additionally, a main effect of angle was revealed, *F*_(2, 52)_ = 23.491, *p* < 0.001, $\eta_{\text{p}}^{2}$ = 0.475, with participants across groups performing worse on trials with small (10–30°) angles of rotation (*M* = 89.58%, *SE* = 1.69%) than either medium (40–60°) rotations (*M* = 98.63%, *SE* = 0.37%) or large (70–90°) rotations (*M* = 97.62%, *SE* = 0.70%). The interaction between angle and group was also significant, *F*_(2, 52)_ = 4.34, *p* = 0.02, $\eta_{\text{p}}^{2}$ = 0.143. Specifically, both groups performed more poorly on small rotation than medium rotation trials (PD: *p* < 0.001; HOA: *p* = 0.04), but only the PD group also performed more poorly on small rotation than large rotation trials (PD: *p* < 0.001; HOA: *p* = 0.07) (Figure 3).

For object discrimination, there was a main effect of group, *F*_(1, 26)_ = 6.37, *p* = 0.02, $\eta_{\text{p}}^{2}$ =.197, with the HOA group (*M* = 99.45%, *SE* = 0.45%) scoring higher on the task than the PD group (*M* = 97.87%, *SE* = 0.45%). There was no main effect of session. However, the interaction between session and group was significant, *F*_(1, 26)_ = 5.65, *p* = 0.03, $\eta_{\text{p}}^{2}$ = 0.178, with the PD group performing better in the morning than the afternoon, *p* = 0.02, but no corresponding difference in the HOA group, *p* = 0.45.

For face discrimination, the results also showed a main effect of group, *F*_(1, 26)_ = 10.71, *p* < 0.01, $\eta_{\text{p}}^{2}$ = 0.292, with the HOA group (*M* = 95.39%, *SE* = 1.51%) scoring higher on the task than the PD group (*M* = 88.39%, *SE* = 1.51%). No other effects or interactions were significant.

**Figure 4:**
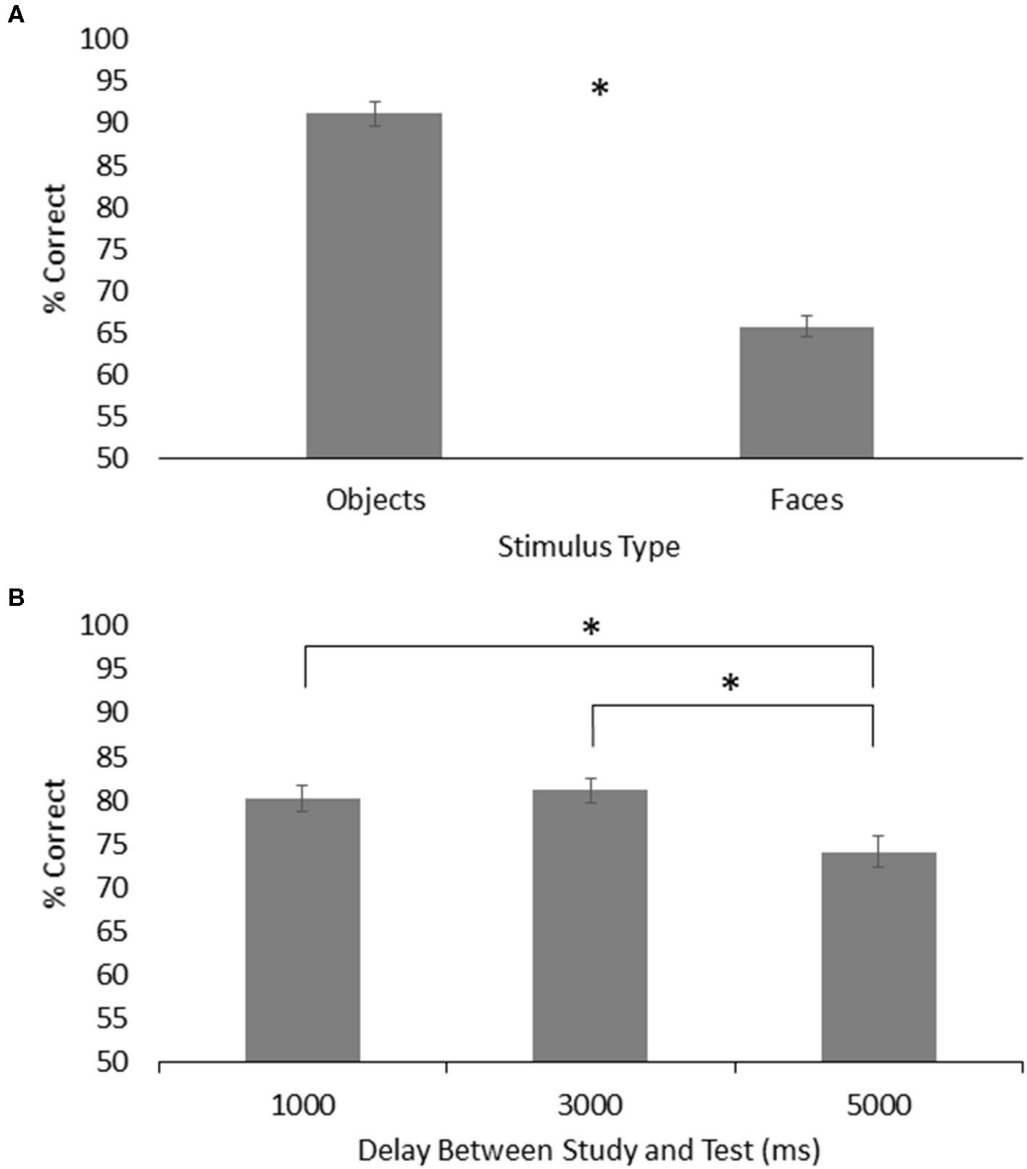
**(A)** Mean and standard error for memory of objects and faces across both groups on the visual working memory task. **(B)** Mean and standard error for visual working memory across both groups for each time delay. Asterisks designate significance at *p* < 0.05.

For visual working memory, the results showed a main effect of group, *F*_(1, 26)_ = 4.62, *p* = 0.04, $\eta_{\text{p}}^{2}$ = 0.151, with the HOA group (*M* = 80.75%, *SE* = 1.50%) scoring higher than the PD group (*M* = 76.19%, *SE* = 1.50%). There was a main effect of stimulus type, *F*_(1, 26)_ = 224.20, *p* < 0.001, $\eta_{\text{p}}^{2}$ = 0.896, with participants across groups performing better on object trials (*M* = 91.17%, *SE* = 1.47%) than face trials (*M* = 65.77%, *SE* = 1.24%). There was also a main effect of delay, *F*_(2, 52)_ = 6.98, *p* < 0.01, $\eta_{\text{p}}^{2}$ = 0.212, with participants across groups performing better on both the 1,000 ms delay trials (*M* = 80.21%, *SE* = 1.57%) and 3,000 ms delay trials (*M* = 81.10%, *SE* = 1.30%) than on the 5,000 ms delay trials (*M* = 74.11%, *SE* = 1.84%) (*p* = 0.04 and *p* = 0.01, respectively) (Figure 4). No other effects or interactions were significant.

**Figure 5:**
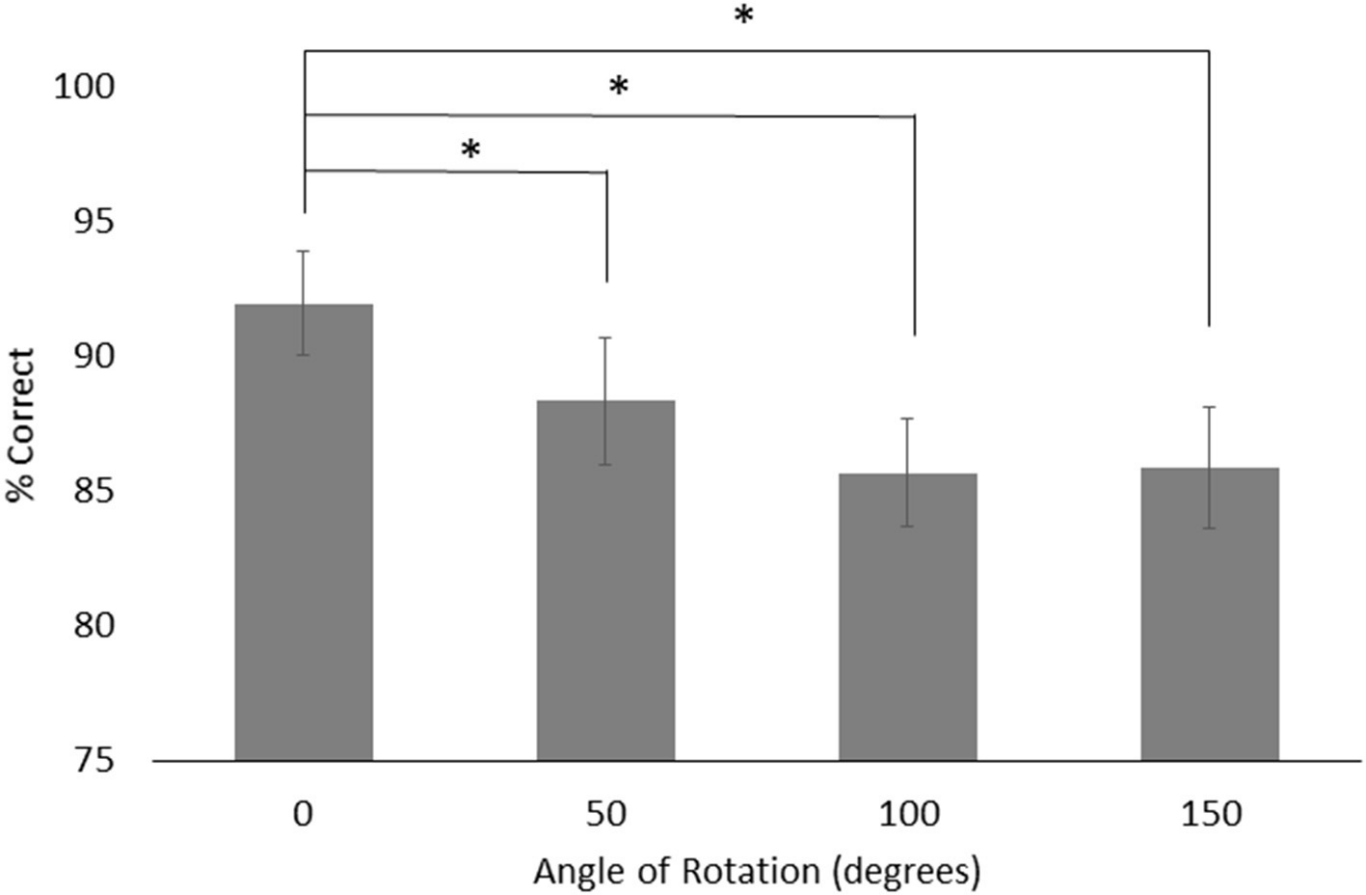
Mean and standard error for object rotation across both groups for each angle of rotations. Asterisks designate significance at *p* < 0.05.

For object rotation, there was a main effect of group, *F*_(1, 24)_ = 12.043, *p* < 0.01, $\eta_{\text{p}}^{2}$ = 0.334, with the HOA group making more correct judgments (*M* = 93.63%, *SE* = 2.31%) than the PD group (*M* = 82.32%, *SE* = 2.31%). There was also a main effect of rotation angle, *F*_(3, 72)_ = 3.25, *p* = 0.03, $\eta_{\text{p}}^{2}$ = 0.119. Both groups performed better on trials with 0° of rotation (*M* = 91.99%, *SE* = 1.96%) than trials with 50° (*M* = 88.36%, *SE* = 2.38%), 100° (*M* = 85.68%, *SE* = 2.00%), or 150° (*M* = 85.90%, *SE* = 2.26%) of angular rotation (*p* = 0.02, *p* = 0.02, and *p* = 0.03, respectively) (Figure 5). No other effects or interactions rose to the level of significance.

### Motor UPDRS

A within subjects *t*-test showed a significant effect of levodopa administration, *t* = −3.08, *p* < 0.01, with symptoms being more severe in the morning (*M* = 26.86, *SD* = 13.83) than in the afternoon (*M* = 22.12, *SD* = 11.38). However, there were no significant associations between the change in motor UPDRS score and change in any visual processing task (Line Discrimination, *p* = 0.94; Object Discrimination, *p* = 0.86; Facial discrimination, *p* = 0.86; Visual Working Memory, *p* = 0.71; Object Rotation, *p* = 0.63).

## Discussion

The purpose of this study was to examine cognitive visual processing in individuals with PD and how resulting symptoms respond to levodopa. The aims were to determine where in the pathway visual processing might be breaking down and the extent to which performance on visual tasks varied between ON-medication and OFF-medication states. As anticipated, the PD group performed significantly worse than the HOA group across all tasks. However, performance deficits in the PD group were unaffected by the administration of levodopa, suggesting that mechanisms other than dopamine depletion may be responsible for impairments in cognitive vision. Further, given that there were no dramatic changes in mean difference between groups across tasks, the implications for where in the pathway visual processing is breaking down requires a somewhat nuanced interpretation.

### Differences Between Groups, but Not Medication, Why?

The striatum in the basal ganglia has the highest expression of dopamine receptor mRNA (both D_1_ and D_2_) of any region of the human brain (17) and dysfunction of dopaminergic signaling in the striatum is believed to be the principal cause of the majority of motor and cognitive deficits in PD (18). Thus, to the extent that visual tasks recruit processing resources from the basal ganglia, it was expected that dopamine depletion in the basal ganglia associated with PD would lead to deficits in the visual processing tasks used in this study. The results of this study did show that persons with PD performed worse on cognitive vision tasks as compared to healthy older adults, which may support this notion. However, there were no significant improvements in performance after the administration of levodopa across all tasks. This would suggest that other mechanisms may be involved in cognitive vision impairment in persons with PD.

More recent research indicates that the pathways between the basal ganglia and the visual pathway are involved in the learning aspects of processing visual stimuli. Specifically, the occipitotemporo-neostriatal pathway has been implicated in learning visual associations based on reward, such that damage does not lead to impairments in fundamental visual discrimination of stimuli (19). In contrast, the occipitotemporo-ventral striatum pathway has been implicated in the assignment of value to stimuli (20). This finding has been supported by imaging studies of humans indicating that the ventral striatum shows selective activation for rewarded outcomes (21). Taking together, this evidence suggests PD pathology in the basal ganglia would be more likely to disrupt learning of object associations and values and may explain the lack of medication effect on the object discrimination tasks in this study. This finding is in keeping with previous research. Lange et al. found that dopaminergic modulation had no significant effect on performance of spatial or pattern recognition memory tasks, simultaneous or delayed match-to-sample tasks, or visual associative learning tasks (13). Moreover, Pillon et al. found no difference in performance on a ventral pathway-dependent visual task when individuals were in on vs. off medication states (22). Thus, the lack of dopaminergic modulation on the high level cognitive vision tasks (e.g., object discrimination) in this study may suggest that other non-dopaminergic mechanisms underlie the control of these tasks, but are nonetheless still affected by PD pathology.

The significant difference between persons with PD and HOAs across all tasks revealed in this study may suggest that impairments in high level cognitive visual tasks are simply the result of deficits in more basic visual processing that are not dependent upon dopaminergic activity. For instance, researchers have reported symptoms such as decreased blink rate, dry eyes, blepharospasm (uncontrolled spastic movements of the eyelids and brows, bilaterally) that are linked to ocular surface irritation in persons with PD (3). Other researchers have also reported the presence misfolded and phosphorylated α-synuclein in the retinas of individuals with PD (4). Together, these symptoms may account for at least some of the acuity and contrast sensitivity deficits observed in PD that may be unrelated to dopamine. Alternatively, PD may lead to impaired dopaminergic signaling in areas of the visual system without any direct involvement of the basal ganglia. Dopamine receptors have been observed in a wide range of visual areas involved in object identification and recognition, including the retina (23), lateral geniculate nucleus, primary visual cortex, and prefrontal cortex (16). In the human retina, dopamine function is thought to contribute to light adaptation and establishing the center-surround organization of retinal receptive fields that facilitates edge detection (24, 25). Moderate to high levels of D_2_ receptor mRNA were identified in the lateral geniculate nucleus, while moderate to high levels of D_1_ receptor mRNA were identified in the primary visual cortex (V1) (16). Thus, dopamine depletion in these areas might lead to deficits in perception of visual features to which the retinal fields of the lateral geniculate nucleus and primary visual cortex are most responsive (e.g., bars of light of particular lengths, either stationary, or moving perpendicular to their long axes) (26). These lower level visual deficits may then affect downstream processing of high level cognitive vision.

A second explanation for the differences between PD and HOA groups, but no improvement with levodopa may be due to diffuse neural atrophy. For instance, studies have shown that PD is associated with microstructural changes in both gray and white matter that appear to scale with disease severity (27), and may be associated with mild cognitive impairment (27, 28). Moreover, the changes in gray matter volume are in regions that have implications for visual processing (9). Patients with PD who have developed dementia also show decreased volume in the occipital cortex (29). Koh et al. (30) found that abnormal stereopsis in individuals with PD was associated with significantly reduced gray matter in the extrastriate visual cortex. Pereira et al. (9) found that decreased performance on a face recognition task was associated with decreased gray matter volume in the fusiform gyrus, parahippocampus, middle occipital gyrus, and inferior frontal gyrus in persons with PD. Decreased performance on a visual form discrimination task was associated with gray matter losses in the superior parietal and occipital lobes, as well as in the inferior and middle frontal gyri. Finally, decreased performance on a test of memory for faces was associated with gray matter losses in the right parahippocampus (9). It is therefore likely that at least some of the visual deficits observed in this study may also be at least partially accounted for by neural atrophy.

Finally, if deficits in basic visual processing in this study are dependent on dopaminergic processing, there remains the question of why dopaminergic medication did not improve performance. Research has shown that the basal ganglia has fairly robust mechanisms to compensate for the loss of dopamine in PD, and as a result symptoms of dopamine depletion in the basal ganglia which lead to a clinical diagnosis of PD are not likely to be observed in the early stages of pathology or injury (31). If dopamine circuitry in the visual system lacks similar compensatory mechanisms, it may be that visual symptoms become refractory to treatment with dopamine precursor medications while the same drugs remain efficacious for basal ganglia-dependent pathology.

### Breakdown in the Visual Processing Pathway

This study was designed to employ tasks that could provide evidence concerning which areas the visual system might be the source of any observed visual impairment. Specifically, each of the five visual tasks in the current study have been found in previous studies to recruit a slightly different constellation of visual areas. If PD pathology had disproportionately affected any of these areas, we would have predicted that some appreciable number of individuals in the PD group would have performed at or near chance for tasks that required processing in this area while all members of the HOA group remained comparatively unaffected. For example, if the prefrontal cortex deteriorated significantly in PD while the ventral visual pathway itself was largely spared, we would predict that members of the PD group would perform at or near chance on the visual working memory and object rotation tasks while showing little or no deficit on the first three discrimination tasks. On the other hand, if PD pathology was specific to early visual areas such as the retina or V1, we would expect members of the PD group to perform close to chance on all five tasks.

However, no dramatic drop to chance level was observed for the PD group for any task. Instead, the PD group performed significantly worse than the HOA group on all tasks while never showing a profound deficit on any one task. One way to account for the pattern of results observed in this study would be to postulate that two regions critical for these tasks were particularly susceptible to PD pathology: one in the early visual system and one in motor cortical regions. The former would account for the small but persistent group differences observed in the first four tasks. It may simply be that whatever damage produced the impaired fine visual discrimination observed in the line discrimination task produced a similar impairment in the discrimination of the object and face stimuli used in this study. On the other hand, impairment of the motor cortical regions would account for the increase in effect of group observed in the object rotation task. This position was supported by research suggesting a role for motor cortical areas in mental simulation and emulation (32, 33) and research indicating that these areas show altered or diminished function in PD (34, 35). However, no brain imaging techniques were used in this study to confirm the role of the motor cortex in visual impairments in persons with PD.

### Limitations

There are limitations to this study. Participants were not screened for motor fluctuations. Some participants may have been under the influence of a long-duration levodopa benefit, which may obscure the effect of levodopa on the visual tasks. While the 12 h wash out is a common practice in previous PD research, 12 h may not have been long enough to fully washout the effects of levodopa or any additional medication participants were taking. These limitations may explain the lack of effect of levodopa on the visual tasks in this study. However, it is important to note that there was still an effect of levodopa on UPDRS motor symptoms. The wash out period or long-duration benefit may affect visual symptoms differently than motor symptoms. In addition, since cognitive testing was not completed, the contribution of cognitive impairment to impairments in cognitive vision in this sample cannot be ruled out. Finally, the sample size was small, which limits the generalizability of the results.

## Conclusion

This study was designed to investigate the effects of dopamine and dopaminergic medications on impairments to high-level vision in PD. Despite the key role that dopamine depletion plays in much of the pathology of PD, the results of the present study provide evidence that modulation of dopamine levels may not improve or impair cognitive vision in individuals with PD, which represents a novel finding in visual research. These results indicate that continued research is needed to fully understand vision impairments and the most beneficial therapeutic strategy to treat this impairment in persons with PD.

## Data Availability Statement

All datasets generated for this study are included in the article/supplementary material.

## Ethics Statement

The studies involving human participants were reviewed and approved by Iowa State University Institutional Review Board. The patients/participants provided their written informed consent to participate in this study.

## Author Contributions

SA and ES: conception and execution of the research project, design, review and critique of the statistical analysis, and review and critique of the manuscript. SA: organization and execution of the research project, execution of the statistical analysis, and writing of the first draft of the manuscript. Both authors contributed to the article and approved the submitted version.

## Conflict of Interest

The authors declare that the research was conducted in the absence of any commercial or financial relationships that could be construed as a potential conflict of interest.
